# Efficiency Improvement of the Clinical Pathway in Cardiac Monitor Insertion and Follow-Up: Retrospective Analysis

**DOI:** 10.2196/67774

**Published:** 2025-03-21

**Authors:** Ville Vanhala, Outi Surakka, Vilma Multisilta, Mette Lundsby Johansen, Jonas Villinger, Emmanuelle Nicolle, Johanna Heikkilä, Pentti Korhonen

**Affiliations:** 1 Tampere Heart Hospital, Elämänaukio 1, Tampere, 33520, Finland, 358 3311716; 2 Jamk University of Applied Sciences, Jyväskylä, Finland; 3 Medtronic Denmark, Copenhagen, Denmark; 4 Medtronic International Trading Sarl, Tolochenaz, Switzerland

**Keywords:** insertable cardiac monitor, clinical pathway, nurse-led service, task shifting, efficiency improvement, remote monitoring

## Abstract

**Background:**

The insertable cardiac monitor (ICM) clinical pathway in Tampere Heart Hospital, Finland, did not correspond to the diagnostic needs of the population. There has been growing evidence of delegating the insertion from cardiologists to specially trained nurses and outsourcing the remote follow-up. However, it is unclear if the change in the clinical pathway is safe and improves efficiency.

**Objective:**

We aim to describe and assess the efficiency of the change in the ICM clinical pathway.

**Methods:**

Pathway improvements included initiating nurse-performed insertions, relocating the procedure from the catheterization laboratory to a procedure room, and outsourcing part of the remote follow-up to manage ICM workload. Data were collected from electronic health records of all patients who received an ICM in the Tampere Heart Hospital in 2018 and 2020. Follow-up time was 36 months after insertion.

**Results:**

The number of inserted ICMs doubled from 74 in 2018 to 159 in 2020. In 2018, cardiologists completed all insertions, while in 2020, a total of 70.4% (n=112) were completed by nurses. The waiting time from referral to procedure was significantly shorter in 2020 (mean 36, SD 27.7 days) compared with 2018 (mean 49, SD 37.3 days; *P*=.02). The scheduled ICM procedure time decreased from 60 minutes in 2018 to 45 minutes in 2020. Insertions performed in the catheterization laboratory decreased significantly (n=14, 18.9% in 2018 and n=3, 1.9% in 2020; *P*=<.001). Patients receiving an ICM after syncope increased from 71 to 94 patients. Stroke and transient ischemic attack as an indication increased substantially from 2018 to 2020 (2 and 62 patients, respectively). In 2018, nurses analyzed all remote transmissions. In 2020, the external monitoring service escalated only 11.2% (204/1817) of the transmissions to the clinic for revision. This saved 296 hours of nursing time in 2020. Having nurses insert ICMs in 2020 saved 48 hours of physicians’ time and the shorter scheduling for the procedure saved an additional 40 hours of nursing time compared with the process in 2018. Additionally, the catheterization laboratory was released for other procedures (27 h/y). The complication rate did not change significantly (n=2, 2.7% in 2018 and n=5, 3.1% in 2020; *P*=.85). The 36-month diagnostic yield for syncope remained high in 2018 and 2020 (n=32, 45.1% and n=36, 38.3%; *P*=.38). The diagnostic yield for patients who had stroke with a procedure in 2020 was 43.5% (n=27).

**Conclusions:**

The efficiency of the clinical pathway for patients eligible for an ICM insertation can be increased significantly by shifting to nurse-led insertions in procedure rooms and to the use of an external monitoring and triaging service.

## Introduction

### Background

Insertable cardiac monitors (ICMs) are indicated for long-term monitoring of heart rhythms, primarily for the indications of unexplained syncope and cryptogenic stroke (CS) or transient ischemic attack (TIA) [1-4]. For patients monitored with an ICM, a remote monitoring system transfers ICM data daily to the hospital staff for analysis. The 2023 European Heart Rhythm Association–Heart Rhythm Society expert consensus on remote monitoring recommends remote monitoring as standard of care for ICMs [5]. However, remote monitoring can create a significant data burden [6], which can be challenging in the current context of clinical staff shortage and disparities between different populations for access to services [7]. Recent studies have indicated that the in-office time to follow-up an ICM patient took approximately 39.9 minutes of staff time, while remote follow-up required only 11.3 minutes [8]. In addition, in studies regarding nurse-led ICM service, it has been confirmed that in an outpatient setting, ICM service by specially trained nurses can lead to significant savings without compromising the safety of the procedure [6].

Workforce challenges are well-known across countries. Therefore, the 2023 European Heart Rhythm Association–Heart Rhythm Society consensus statement recommends the effective management of remote monitoring clinics to focus on adequate staffing with clear roles and responsibilities, on-going staff education, and efficient high-priority alert systems [5]. Nurse-led services play a particularly important role for efficient ICM services, as international case studies show that nurses can conduct both ICM insertions and remote follow-up effectively and safely [9].

Additionally, the use of third-party resources can be an opportunity to efficiently manage remote monitoring of ICM patients and a solution for dealing with increased device clinic volume [8,10]. ICMs are prone to produce a heavy workload for the remote monitoring clinic (25% of all transmissions, 10 times more frequent than for a pacemaker) [11].

In Finland, health services are challenged due to the shortage of trained health care professionals and resources. For example, Finland has fewer cardiologists than the average for the member countries of the European Society of Cardiology (ESC; Finland 50.5 per million people vs ESC countries 85.1 per million people) [7]. Finland also faces a growing need for nurses in Finland [12]. The Finnish government has launched the “Good Work Program” to ensure the sufficiency and availability of personnel in health care, social welfare, and rescue services. The program aims to increase the attractiveness of working within the social and health care sector by developing the structures and clarifying the tasks between the personnel [13].

At the Finnish Tampere Heart Hospital, both insufficient staff resources and a growing number of patients in need of ICM monitoring led to the restructuring of the clinical patient pathway. The changes centered around training nurses to perform ICM insertions, the inclusion of the neurology department in patient pathways, moving the remaining ICM procedures out of the catheter laboratory, and the use of third-party triaging services.

However, the impact of these changes from the perspective of efficient resource management and quality of care is unknown. Thus, we conducted an analysis of the changes in clinical pathways at the Tampere Heart Hospital, assessing the impact on patient pathway efficiencies and the quality of care.

### Analyzing the ICM Pathway in 2018

**Figure 1.**
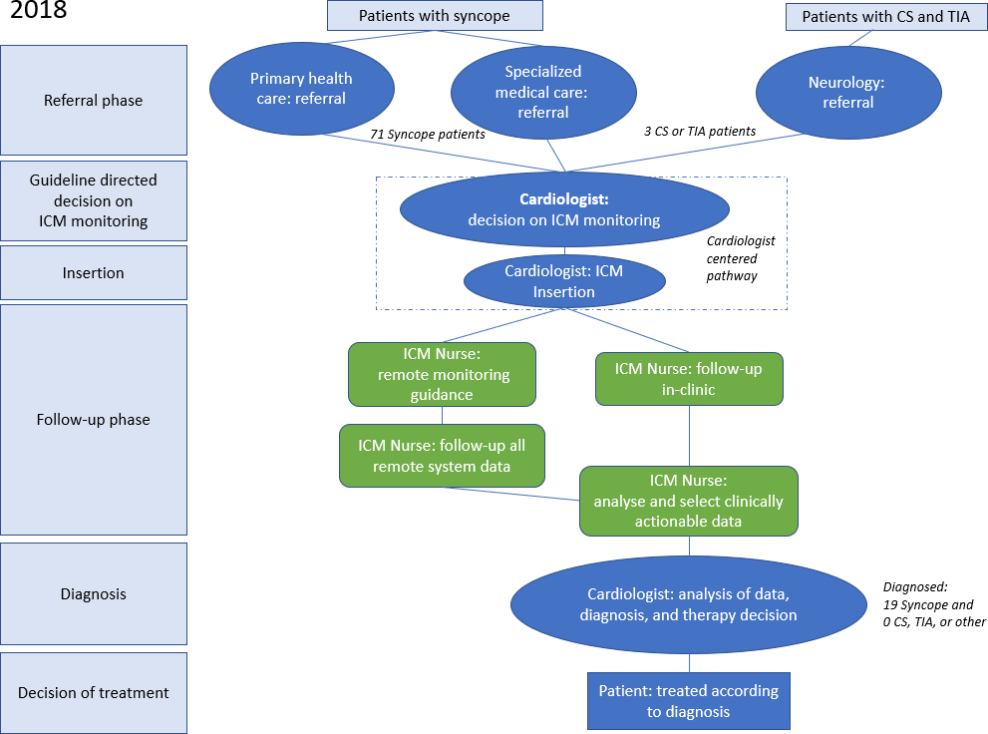
Patient pathways in 2018. CS: cryptogenic stroke; ICM: insertable cardiac monitor; TIA: transient ischemic attack.

In 2018, the Tampere Heart Hospital analyzed the prevailing ICM clinical pathway, and the way tasks were divided between professionals in each phase. The 2018 patient pathway was characterized by cardiology-centric decision-making for ICM insertions. Only a few patients who had CS were referred to the cardiology department even though the neurologist could make a referral to atrial fibrillation (AF) monitoring therapy for secondary prevention of CS and TIA. At the time, the ESC guidelines for AF management from 2016 were valid [3]. Unexplained patients who had syncope were referred by a general practitioner or the emergency department doctor to a cardiology clinic, where a cardiologist assessed whether these patients required an ICM based on the ESC guidelines from 2018 [1]. If an ICM was recommended for CS, TIA, or unexplained syncope, the patient was placed on a waiting list for the procedure and later invited to an outpatient clinic for device insertion by a cardiologist in a catheterization laboratory (Figure 1). The laboratory time was a highly demanded resource for performing more advanced interventional cardiological procedures.

### Changes in the ICM Pathway as of 2020

#### Increasing Access to ICM Monitoring for Patients Who Had CS or TIA

**Figure 2.**
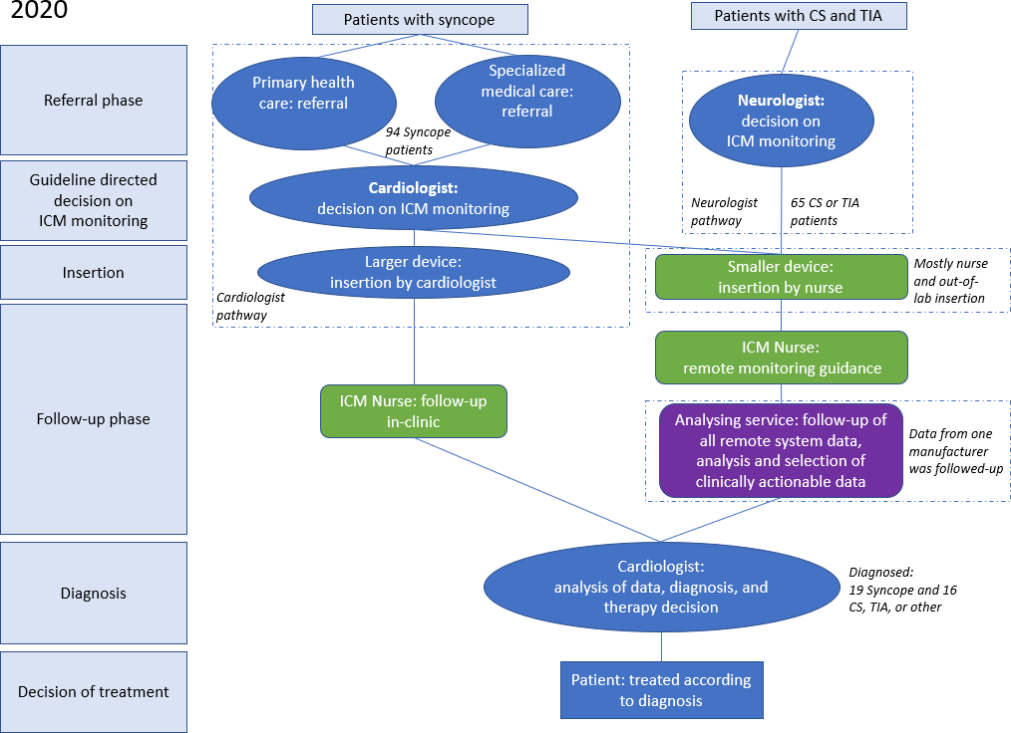
Patient pathways in 2020. CS: cryptogenic stroke; ICM: insertable cardiac monitor; TIA: transient ischemic attack.

Based on the analysis, the clinical pathway was changed to improve its efficiency. The referral via cardiologist was a barrier for ICM monitoring for patients who had CS or TIA. To increase the access of patients who had CS, the neurologist could refer patients directly to an ICM procedure (Figure 2). Therefore, the decision on ICM insertions was transferred to the neurologist. This was in line with the updated 2020 ESC guidelines for AF management which had a stronger recommendation for ICM insertions for patients who had CS.

#### Increasing Patients’ Access to ICM Insertion Through Nurse-Inserted ICM in the Procedure Room

The initial change focused on solutions for increasing the ICM insertion capacity of the hospital as well as patients’ access to diagnostic services. Drawing from experiences abroad [6,9,14], where nurses safely and effectively conducted ICM insertions, the conclusion was made that training nurses to perform ICM procedures was safe and feasible.

The first ICM nurse-led insertion training program was initiated in Finland in 2019. The content was designed corresponding to the international, “nonphysician insert” ICM training program [6]. On the organizational level, the trained specialized nurses were deemed comparable to advanced practice providers as defined in international literature and publications [9]. Registered nurses underwent specialized training to perform ICM insertions (Multimedia Appendix 1). Based on the training and monitoring of 5 patients’ ICM insertions under the supervision of a cardiologist, the Tampere Heart Hospital authorized 3 nurses to perform independent ICM insertions, thus officially delegating some of the physicians’ responsibilities to the nurses officially to redistribute the workload.

Limited availability of the catheterization laboratory and management of the patient who had ICM workflow in the hospital led to launching nurse-led ICM insertions in a clean follow-up room specifically equipped for this procedure. The improved ICM clinical pathway with nurses performing ICM insertion of smaller devices was launched in the beginning of 2020. Larger ICMs were still on the market as well and cardiologists implanted them (Figure 2).

#### Outsourcing ICM Data Monitoring and Triaging

Another notable change pertained to managing the workload associated with ICM data, as most ICMs were monitored remotely. Considering that a significant portion of the data were not clinically actionable and given the limitations in staff time, it was decided to outsource the first line analysis and triaging of remote follow-up data (Figure 2). The external monitoring service (FocusOn, Medtronic), consisting of technicians and rhythm cardiology professionals, analyzed the electrocardiogram data from patients who had ICM. They determined the urgency of the information and conveyed it to the hospital. This approach enables efficient data management, allowing hospital staff to focus on patients needing immediate attention [15] or perform additional ICM insertions.

## Methods

### Efficiency Assessment

A retrospective registry study was performed to assess the impact of the pathway changes. We computed key efficiency and safety metrics for the Tampere Heart Hospital before (2018) and after (2020) the change in the clinical pathways. Efficiency metrics included the number of patients treated with ICMs for unexplained syncope and CS or unexplained TIA, the number of ICM insertions performed by nurses and cardiologists, procedure time, the number of insertions carried out in the catheterization laboratory, waiting time, diagnostic yield, and time to diagnosis. Clinically significant arrhythmia (bradycardia or tachycardia) was included in the diagnostic yield for patients who had syncope. For patients who had stroke, the diagnostic yield was measured as the proportion of patients with AF >6 minutes. Safety measures included the number of infections.

### Patient Population and Data Collection

Data collection encompassed all consecutive patients who had ICM at the Tampere Heart Hospital, irrespective of their indications, in the years 2018 and 2020. The data collection process was established as part of the clinic’s ongoing medical care quality improvement efforts. Data were retrospectively collected from the patient records and procedure registry and identified using procedure codes and device serial numbers.

### Ethical Considerations

This study followed the ethical principles of the Declaration of Helsinki. Tampere University Hospital's Research Services of the Wellbeing services county of Pirkanmaa provided the permissions for the patient-level data collection from the electronic health record (R23641X). Because patients weren't contacted directly, informed consent wasn't required according to Finnish law. To protect patient privacy, patients who had ICM-level data were pseudonymized and subsequently aggregated into an anonymized format to prevent the identification of individuals. The data were handled according to the General Data Protection Regulation policy of the European Union.

### Statistical Analysis

Descriptive tabling of the quantitative variables was performed in Excel (version 2302; Microsoft 365 apps for enterprise). For categorical variables, the chi-square test was used to compare the distributions of 2 or more groups. For continuous variables, a 2-tailed *t* test was conducted to test for statistically significant differences. All calculations were carried out according to the intention to treat principle.

## Results

### Participants

In 2018, 74 consecutive patients were included in this study and in 2020, it was 159.

The proportion of female patients was 43.2% (n=32) and 51.6% (n=82) in 2018 and 2020, respectively. As they were being treated in an adult cardiology department, all patients were over 16 years of age. Most of the patients were aged between 40 and 79 years (n=58, 78.3%) in 2018, with a similar age distribution in 2020 (n=114, 71.7%). The median age of the patients was 66 (55.5-76.8) years in the 2018 patient population and 67 (54.0-75.0) years in the 2020 population. Participants’ characteristics are presented in Table 1.

**Table 1.** Characteristics of participants who received ICM^a^ insertions in 2018 and in 2020.

		2018 (n=74), n (%)	2020 (n=159), n (%)	*P* value
Sex (female)		32 (43.2)	82 (51.6)	.24
Age (years)				.35
	16‐39	4 (5.4)	24 (15.1)	
	40‐59	22 (29.7)	33 (20.8)	
	60‐79	36 (48.6)	81 (50.9)	
	80+	12 (16.2)	21 (13.2)	

a ICM: insertable cardiac monitor.

### Use of ICM According to Guidelines

In 2018, the indication for ICM insertion was mainly unexplained syncope (n=71, 95.9%) with 2.7% (n=2) of the patients indicated with CS. In contrast, in 2020, a total of 59.1% (n=94) were indicated with unexplained syncope and 39% (n=62) with CS. The number of patients receiving ICMs increased substantially from 2018 to 2020 (*P*<.001). For patients who had syncope, the increase was from 71 to 94. Notably, the use of ICMs in patients with SC or TIA substantially increased from 2018 (2 patients) to 2020 (62 patients; Table 2).

**Table 2.** Results—change in clinical pathway and safety.

		2018	2020	*P* value
Indication, n (%)				<.001
	Indication syncope	71 (95.9)	94 (59.1)	
	Indication cryptogenic stroke or TIA^a^	2 (2.7)	62 (39)	
	Other	1 (1.4)	3 (1.9)	
Waiting time to procedure (day), mean (SD)		49 (37.3)	36 (27.7)	.02
Nurse insertions, n (%)		0 (0)	112 (70.4)	<.001
Scheduled procedure time (min), n		60	45	
Insertion in catheterization laboratory, n (%)		14 (18.9)	3 (1.9)	<.001
Overall complication rate, n (%)		2 (2.7)	5 (3.1)	.85
Data burden, n (%)				<.001
	Patients on remote monitoring	38 (51.3)	108 (67.9)	
	Patients on analyzing service	0 (0)	108 (67.9)	

a TIA: transient ischemic attack.

### Waiting Time

A 2-sample *t* test was performed to compare the average waiting time from referral to insertion in 2018 and 2020. The average waiting time decreased significantly from 49 days in 2018 to 36 days in 2020 (*P*=.02; Table 2).

### Resource Use

In 2018, physicians conducted all insertions, while in 2020, 70.4% (n=112) of the ICM insertions were performed by specially trained nurses. The number of inserted ICMs doubled from 74 in 2018 to 159 in 2020. Delegating the responsibility of ICM insertions to trained nurses allowed physicians to allocate their time to other essential procedures and interventions. This transition to nurse-performed insertions in 2020 resulted in a saving of 48 hours (more than 6 working days) of physicians’ time, a noteworthy improvement from the process in 2018 (Table 2).

### Catheterization Laboratory Use

In 2018, 18.9% (n=14) of the insertions were completed in the catheterization laboratory, whereas in 2020, this figure was reduced to 1.9% (n=3; *P*<.001). Additionally, the scheduled procedure time for ICM insertion decreased from 60 minutes in 2018 to 45 minutes in 2020. The streamlined procedure scheduling saved an additional 40 hours (1 wk) of nursing time and released the catheterization laboratory for other critical procedures, amounting to 27 hours per year (Table 2).

### Safety and Quality of the Procedure

All procedure-related complications were collected. The procedure-related complications were pain (1 patient in 2020), infection (2 patients in 2020), bleeding (2 patients in 2020), and device migration (1 patient in 2020). A total of 4 ICMs were explanted due to complications (3 relating to infection and 1 relating to pain). The complication rate remained consistent, with no significant change, at 2.7% (n=2) in 2018 and 3.1% (n=5) in 2020 (*P*=.85).

R-wave sensing data were only registered in 2020 after the initiation of nurse insertions. The average R-wave at implant in 2020 was 0.57 (SD 0.3) mV with 8 (5%) patients having an R-wave below 0.2 mV.

### Nurse Productivity

Remote monitoring was set up for 51.3% (n=38) of the patients in 2018 and for 67.9% (n=108) in 2020. In 2018, none of the remote-monitored patients who had ICM were followed up by an outsourced analyzing service, while in 2020, all ICM remote-monitored patients (n=108) were in the FocusOn-system. In 2018, nurses were responsible for analyzing all remote transmissions, consuming a substantial amount of their time. The number of transmissions that needed analyzing from nurses was not available. In 2020, the initial review and triaging of remote transmissions were outsourced to an external monitoring center. This external service escalated 11.2% (204 out of 1817) of the transmissions to the clinic for review. Assuming an average of 11 minutes per transmission by a nurse [8,10,16], this external service saved 296 hours (approximately 40 working days corresponding to almost 2 mo) of nursing time in 2020 (Table 2).

### Diagnostic Yield

Notably, the quality of the diagnostic pathway was high, with a high diagnostic yield despite the increase in inserted ICMs from 2018 to 2020 (Table 3). The 1-year diagnostic yield for patients with syncope remained high and exhibited no statistically significant difference between 2018 and 2020 (n=19, 26.7% vs n=19, 20.2%; *P*=.32). The 36-month diagnostic yield for patients who had syncope was generally high, with no statistically significant difference between 2020 (n=36, 38.3%) and 2018 (n=32, 45.1%; *P*=.38). The time to diagnosis was not statistically significantly different in 2018 and 2020 for patients who had syncope (109 vs 114 days; *P=*.88). Further information of detected arrhythmias is included in Multimedia Appendix 2.

**Table 3.** Diagnostic yield-intention to treat (2018: n=74; 2020: n=159).

	12 month follow-up, n (%)		*P* value	24 month follow-up, n (%)		*P* value	36 month follow-up, n (%)		*P* value
	2018	2020		2018	2020		2018	2020	
Overall	19 (25.7)	35 (22)	.54	31 (41.9)	52 (32.7)	.17	32 (43.2)	63 (39.6)	.60
Syncope	19 (26.7)	19 (20.2)	.32	31 (43.7)	31 (33)	.16	32 (45.1)	36 (38.3)	.38
Stroke	0 (0)	17 (27.4)	N/A^a^	0 (0)	21 (33.9)	N/A	0 (0)	27 (43.5)	N/A

a N/A: not applicable.

The 1-year diagnostic yield (AF diagnosis) for patients who had CS was 27.4% (n=17) and the 36-month diagnostic yield was 43.5% (n=27) in 2020. The average time to diagnosis for patients who had stroke was 127 days in 2020.

## Discussion

### Principal Findings

Our study illustrated that the shift from physician-led ICM insertions to a clinical pathway where nurses inserted the majority of ICMs released a substantial amount of staff time and resources without compromising the quality of the clinical pathway. The efficiency assessment showed that nurse insertion and the use of an external monitoring and triaging service significantly improved the use of hospital resources, such as patient access to ICM insertion, follow-up, and diagnosis. The results correspond to findings from the UK’s National Health Service health care system, where trained nurses have independently been taking care of ICM insertions and follow ups with high quality treatment and safety since 2015 [6].

Regarding the patient follow-up, while in 2018 nurses analyzed all remote monitoring data, in 2020 that part of the workflow was outsourced to an external monitoring and triaging service. As nurses in 2020 monitored only those remote transmissions that were escalated, they could perform more ICM insertions and actionable patient follow-ups. Similar efficiency benefits from outsourcing part of the workflow have been reported previously [10,17]. According to Giannola et al [17], the introduction of such service offered efficiency and effectiveness in patient care more safely than when compared with remote follow-up handled solely at hospital level. Outsourcing the management of remote monitoring data has been seen as a key tool for saving staff time [8,18]. In addition, Biundo et al [8] highlighted the need for appropriate staff resources to support patient management activities, including remote monitoring. Considering the heterogeneity in the infrastructure and staff capacity of hospitals managing patients who had ICM, different organizational models should be considered locally to achieve efficient patient management, including outsourcing part of the remote monitoring workflow [15]. Although the use of an outsourced triaging service will add some costs, more efficient use of hospitals resources and increased number of insertions will probably help hospitals to reclaim the costs from the health care funding system.

Our study at the Tampere Heart Hospital showed both a decrease in the waiting time for the procedure and an increase in the number of patients receiving care in response to the implemented changes. Overall, the number of ICM insertions in 2020 doubled, with indications for CS and TIA also increasing significantly from 2018 to 2020.

The new workflow enabled nurses to gain new skills and broader responsibilities, while physicians could refocus on specialized care. Additionally, the shorter procedure released overall staff time in 2020 compared with 2018. In this study, we only had access to scheduled procedure time and not the actual procedure time. However, these results correspond to the findings of Lim et al [6] with the study conducted in the National Health Service.

In addition, the Tampere Heart Hospital catheterization laboratory was released for other procedures, as the insertions performed in this setting decreased significantly. Rogers et al [16] showed similar results for insertions performed outside the catheterization laboratory. Moving the procedure to office settings saved time spent by patients in hospital, space and resources used, clinical staff time, and, thus, the total costs of the procedure [16]. When aiming to increase efficiency in the clinical pathway, a detailed analysis of all resources supports optimizing the process.

In this study, only cardiac arrhythmia diagnoses were included in the reporting of the diagnostic yield. Furthermore, an “intention to treat” principle was used, hence all patients were included with full follow-up time, even though they were diagnosed, deceased, or exited the population earlier for any other reason.

In our study, the diagnostic yields for patients who had syncope were high both in 2018 and 2020 (n=32, 45.1% and n=36, 38.3%; *P*=.38). In a meta-analysis by Solbiati et al [18], the overall diagnostic yield was reported to be similar to our study (43.9%) [18].

Sanna et al [19] reported the AF detection rate for patients who had stroke to be 12.4% at the 12-month follow-up and 30% at the 36-month follow-up [19]. Our study showed an even higher diagnostic yield of 43.5% (n=27) at 36 months. Notably, the patient population in the initial care pathway only included a very low number of patients who had CS or TIA which prevents a comparison between 2018 and 2020 for this indication [19]. As almost half of the patients who had syncope and patients who had stroke receive a cardiac arrhythmia diagnosis after ICM insertion, there could be underuse of ICMs in both patient groups. There is also a risk for overdiagnosing patients with clinically insignificant arrhythmias and this leading to a potentially harmful therapy (eg, pacemaker implantation after asymptomatic night-time bradyarrhythmia or anticoagulating patient with very short device-detected AF). Choosing patients for ICM insertion is a demanding task and choosing a therapy after device-detected arrhythmia is even more complex. Further studies are needed to address these problems.

Importantly, the changes in the ICM pathway did not compromise patient safety. In this study, the complication rate did not change significantly regardless of whether the procedure was performed solely by a physician in the catheterization laboratory or a procedure room (n=2, 2.7%) or mainly by a nurse in a procedure room (n=5, 3.1%). As the sample size of our study is quite small, even 1 complication will have a significant impact on reported percentages. In earlier studies, procedure-related adverse events have been between 1.1% and 2.6% depending on the location of the procedure [20,21], and the complication rate has been 1% for nurse-performed ICM insertions and 2.2% for physician-performed insertions [6].

At the time of launching this study, there was only 1 other hospital in Finland that had initiated nurse-led insertions. At the time of publishing these results, Finland had 9 hospitals running nurse-led ICM processes. A prospective study assessing the cost-effectiveness of a nurse-led ICM process more precisely could lead to implementing these changes in other health care systems as well.

### Limitations

This study has several limitations. First, it is a single center study with a small number of consecutive patients who had ICM without randomization. Nonetheless, they represent patients from a tertiary level cardiac hospital that serves a population of 520,000 inhabitants [22]. The real-world setting helps to describe how a clinical pathway change is made in practice. Second, the retrospective analysis uses data that was documented or available in the electronic health record. For example, the working time that the nurses used to analyze the data for the 74 patients was not recorded at that time. Therefore, for the efficiency estimation concerning the saved working time of nurses, we used only the 2020 data in comparison with earlier research. Third, R-waves were only measured after the workflow shift to nurse insertions. However, the measured R-wave amplitudes are in line with previously published results [23].

### Conclusions

The change in the clinical pathway to nurse-perfomed insertion in a procedure room and the use of an external monitoring and triaging service significantly improved the efficiency of the pathway for patients indicated for an ICM. In addition, nurse-led insertion released a significant amount of staff time and resources without compromising the quality of the treatment. It can be stated that clinical pathway improvements enable offering ICMs to a greater number of patients to meet the diagnostic demand.

## Supplementary material

10.2196/67774 Multimedia Appendix 1 ICM nurse insertion training program. ICM: insertable cardiac monitor.

10.2196/67774 Multimedia Appendix 2 Arrhythmias detected.

## Abbreviations

AF: atrial fibrillation
CS: cryptogenic stroke
ESC: European Society of Cardiology
ICM: insertable cardiac monitor
TIA: transient ischemic attack
