# Supplementary material for: Efficiency Improvement of the Clinical Pathway in Cardiac Monitor Insertion and Follow-Up: Retrospective Analysis
Source: JMIR Cardio. 2025 Mar 21;9:e67774. doi: 10.2196/67774 (PMC11951822; doi:10.2196/67774)
Supplement: Multimedia Appendix 1 [file cardio-v9-e67774-s001.docx]

A model for nurse insertion training of the cardiac monitors was sought from United Kingdom's national health care system (The National Health Services - NHS). They had been experimenting with the nonphysician insertion of the cardiac monitors since 2015. The quality of insertions had been developed by comparing quality with previous results.

The cardiac monitor nonphysician insertion training structure was designed in 2016 at St Barts Heart Centre (London, UK). First, the nurses watched a training video produced by the device manufacturer, after which the installation of the rhythm monitor was demonstrated in practice. The trainees performed four ICM insertions with an anatomical model of the upper body. Then trainees observed five real procedures, after which each trainee performed five real procedures under the direct supervision of the trainer. After this, five more procedures were performed under the indirect supervision of the trainer.

The Finnish ICM nurse insertion training was designed to correspond to the model proven in St Barts Heart Centre. Before the training, the trainees familiarized themselves with the procedure by watching the insertion video of the cardiac monitor and by taking an online test. Training included practical exercises with an anatomical model of the upper body supervised by the presentative of the device manufacturer. The first trainer on real patients was a consulting cardiologist/electrophysiologist familiar with ICM insertion. After the training course, trainees were required to give a demonstration of their competence in their own hospitals (a cardiologist supervised nurses insertion and gave permission to do nonphysician insertions in the future).

Insertion video used in Finnish ICM nurse insertion training: <https://www.youtube.com/watch?v=ybeYwdPsE2I>

**Table S1.** ICM nurse insertion training course program.

| **Program of ICM Nurse Insertion Training Day 1** |
| --- |
| Introduction of ICM Nurse Insertion Training |
| ICM Indications and technical details |
| ICM Insertion technique  clean room requirements and preparations local anesthesia insertion technique wound closure with tissue glue or stiches potential contraindications |
| ICM insertion and wound closure demo with an anatomical model of the upper body |
| ICM workflow and programming |
| ICM patient education: wound healing, living with ICM remote monitoring |
| Managing ICM process -tips and tricks |
| Benefits of analyzing service |
| Discussion of learning points and wrap up of the day |
| **Day 2 Live Patient Cases** |
| Arrival and dressing with scrubs |
| Patient case 1 and feedback |
| Patient case 2 and feedback |
| Patient case 3 and feedback |
| Lunch |
| Patient case 4 and feedback |
| Patient case 5 and feedback |
| Learning points, feedback and discussion |
| Certificates ceremony |
| Closure of the Nurse Insertion Training |
