# Supplementary material for: Efficiency Improvement of the Clinical Pathway in Cardiac Monitor Insertion and Follow-Up: Retrospective Analysis
Source: JMIR Cardio. 2025 Mar 21;9:e67774. doi: 10.2196/67774 (PMC11951822; doi:10.2196/67774)
Supplement: Multimedia Appendix 2 [file cardio-v9-e67774-s002.docx]

**Table S1.** Diagnoses based on ICM.

|  | Overall | Syncope | Stroke |  | Overall | Syncope | Stroke |
| --- | --- | --- | --- | --- | --- | --- | --- |
|  | 2018 | | |  | 2020 | | |
| ICD-10 code |  | | |  |  | | |
| I44.* (AV-blocks) | 13 % (4) | 13 % (4) | 0 |  | 14% (9) | 17% (6) | 11% (3) |
| I47.* (Tachyarrhythmias) | 19 % (6) | 19 % (6) | 0 |  | 6% (4) | 8% (3) | 4% (1) |
| I48 .* (Atrial fibrillation/flutter) | 13% (4) | 13% (4) | 0 |  | 40% (25) | 25% (9) | 59% (16) |
| I49.* (Sick sinus, SVES, VES) | 50% (16) | 50% (16) | 0 |  | 32% (20) | 42% (15) | 19% (5) |
| Other | 6% (2) | 6% (2) | 0 |  | 8% (5) | 8% (3) | 7% (2) |
| Total | 100% (32) | 100% (32) | 0 |  | 100% (63) | 100% (36) | 100% (27) |
